# Practical approach to the safe use of cannabidiol in patients with refractory epilepsy: a mini review

**DOI:** 10.3389/fphar.2025.1681815

**Published:** 2025-09-04

**Authors:** Cristian E. Navarro

**Affiliations:** School of Medicine, Universidad de Antioquia, Medellín, Colombia

**Keywords:** cannabidiol, cannabis, drug-resistant epilepsy, epilepsy, seizures

## Abstract

Drug-resistant epilepsy (DRE), characterized by seizures that are unresponsive to the use of two or more conventional anti-seizure medication (ASMs), poses a significant therapeutic challenge. In recent years, cannabidiol (CBD), a non-psychoactive compound derived from *Cannabis sativa*, has emerged as a promising adjunctive therapy for specific, severe epilepsy syndromes. The approval of a highly purified 99% CBD oral solution (Epidiolex^®^) has facilitated its integration into clinical practice. However, its effective and safe use requires a nuanced understanding of several practical considerations. This narrative review synthesizes current evidence on the practical aspects of prescribing and administering highly purified CBD oral solution in patients with drug-resistant epilepsy (DRE). I address evidence-based dosing strategies, relevant drug-drug interactions, the profile of adverse-drug reactions (ADRs) and their management, and the critical importance of gradual dose titration. A comprehensive literature search was conducted to identify relevant clinical trials, systematic reviews, observational studies, real-world evidence, and other pertinent publications. A “start low, go slow” dosing approach is essential to mitigate ADRs, which are generally mild to moderate but may include somnolence, decreased appetite, diarrhea, and, most notably, elevated liver transaminases, particularly with concurrent valproate use. Significant pharmacokinetic interactions, especially with clobazam, require careful monitoring and potential dose adjustments of concomitant ASMs. This review offers a consolidated overview of the practical considerations for CBD therapy, aiming to support clinicians in optimizing treatment outcomes for patients with DRE while ensuring patient safety.

## 1 Introduction

Epilepsy is one of the most common neurological disorders globally, affecting millions of people worldwide, especially in low- and middle-income countries (Singh and Sander, 2020; Pacheco-Barrios et al., 2022; Alegría-Muñoz et al., 2025; Feigin et al., 2025). Despite the availability of a wide array of anti-seizure medication (ASMs), approximately one-third of patients with epilepsy continue to experience seizures, a condition known as refractory or drug-resistant epilepsy (DRE) (Kwan and Brodie, 2000; Kwan et al., 2009; Nair, 2016). Persistent seizures in this population are associated with increased risks of morbidity, mortality, cognitive impairment, and reduced quality of life, highlighting the urgent need for novel therapeutic strategies (Devinsky et al., 2018d; Feigin et al., 2025).

In the search for new treatments, considerable attention has focused on the therapeutic potential of cannabinoids, the chemical compounds derived from the *Cannabis sativa* plant. Among these, cannabidiol (CBD) has garnered significant interest due to its anticonvulsant properties without the psychotropic effects associated with delta-9-tetrahydrocannabinol (THC) (Rosenberg et al., 2015). The culmination of preclinical research and clinical trials resulted in the landmark approval of a highly purified 99% CBD oral solution (Epidiolex^®^) for the treatment of seizures associated with Lennox-Gastaut syndrome (LGS), Dravet syndrome (DS), and tuberous sclerosis complex (TSC) (Devinsky et al., 2017; Devinsky et al., 2018a; Devinsky et al., 2018b; Thiele et al., 2018; Thiele et al., 2021). The effectiveness of CBD as an add-on treatment to conventional ASMs has been proven in both children and adults with various etiologies of DRE beyond the syndromes mentioned above, such as cyclin-dependent kinase-like 5 (CDKL5) deficiency disorder, Aicardi syndrome, chromosome 15q11.2–13.1 duplication (Dup15q) syndrome, Doose syndrome, Sturge-Weber syndrome, SYNaptic GTPase Activating Protein (SYNGAP1) encephalopathy, sodium voltage-gated channel alpha subunit 8 (SCN8A)-related epilepsy, epileptic spasms, epilepsy with myoclonic absences, other types of genetic epilepsy, structural epilepsies and even new-onset refractory status epilepticus (NORSE) (Devinsky et al., 2016; Devinsky et al., 2018c; Szaflarski et al., 2018; Kuchenbuch et al., 2020; Gaston et al., 2021; Lattanzi et al., 2021; Patel et al., 2021; Zilmer and Olofsson, 2021; Aydemir and Kandula, 2022; Caraballo et al., 2022; Marchese et al., 2022; Kühne et al., 2023; Navarro, 2023).

The integration of medicinal CBD into the therapeutic armamentarium for DRE marks a significant advancement. However, its safe and effective implementation in clinical practice remains complex. The prescription and administration of CBD require careful consideration of several practical factors to optimize therapeutic outcomes and minimize potential risks. These include determining the appropriate dosage, understanding and managing drug-drug interactions, recognizing and addressing adverse-drug reactions (ADRs), and employing a strategic approach to dose titration (Devinsky et al., 2017; Devinsky et al., 2018a; Devinsky et al., 2018b; Thiele et al., 2018; Thiele et al., 2021; Navarro, 2023). These recommendations apply regardless of patient age and may also be relevant in other diseases where the use of CBD is considered a potential therapeutic alternative (Galvez-Florez et al., 2024; Navarro, 2024; Navarro and Pérez, 2024).

This narrative review aims to provide a comprehensive overview of the practical considerations for the prescription and use of highly purified CBD oral solution in patients with DRE. By synthesizing current evidence from indexed peer-reviewed literature (clinical trials, systematic reviews, observational studies, real-world evidence), I will address key clinical questions regarding dosing regimens, clinically relevant drug-drug interactions, the spectrum of ADRs, and the importance of a gradual dose escalation. Given the widespread perception among physicians of being unprepared to prescribe medicinal cannabis (Evanoff et al., 2017), the aim is to equip clinicians with the necessary knowledge to safely and effectively incorporate oral CBD into the treatment plans for this complex patient population.

## 2 Mechanism of action in epilepsy

The mechanism by which CBD reduces seizure frequency is not yet fully understood. However, CBD has demonstrated affinity for Transient Receptor Potential Vanilloid-1 (TRPV1), the orphan G Protein-Coupled Receptor-55 (GPR55), and the Equilibrative Nucleoside Transporter-1 (ENT-1). TRPV1 and GPR55 are associated with downregulation of pro-inflammatory pathways and calcium influx into mitochondria. ENT-1, through the action of adenosine, enhances anti-inflammatory pathways by inhibiting glutamate neurotransmission (Gray and Whalley, 2020). Additionally, CBD antagonizes the function of the N-methyl-D-aspartate receptor and the P-glycoprotein efflux transporter (Rocha et al., 2020; Boleti et al., 2022).

The modulation in neurotransmission described above may be clinically associated with a significant reduction in interictal discharges (Grayson et al., 2021; Herlopian et al., 2022). Some evidence suggests that CBD-rich extracts may be more effective in reducing seizure frequency than purified CBD alone (Rocha et al., 2020; Navarro, 2023; Navarro, 2024). However, this synergistic effect, known as the entourage effect, between CBD and THC in the treatment of DRE is not fully understood (Schaiquevich et al., 2020).

## 3 Pharmacokinetics of cannabidiol

Absorption: The onset and duration of effect vary depending on the formulation of medicinal cannabis. When smoked, the onset occurs within 3–10 min (Casadiego Mesa and Lastra Bello, 2015), but the duration is shorter compared to orally administered cannabis oil. Absorption via inhalation is dependent on the user’s technique, with only 10%–30% of the compound absorbed through this route (Ingram and Pearson, 2019). In contrast, orally administered CBD reaches maximum concentration within 2.5–5 h, with a half-life of 56–61 h, and a volume of distribution ranging from 20 to 42 L (White, 2019; Landmark and Brandl, 2020). The impact of different oral CBD formulations on the maximum circulating concentration (Cmax) and the time to reach Cmax (Tmax) has been evaluated. Reports indicate that Cmax is higher and Tmax is shorter when oral CBD is administered as a water-soluble formulation (Abbotts et al., 2022).

Due to its lipophilic properties, CBD absorption is enhanced by the intake of a high-fat meals and it tends to accumulate in adipose tissue (Casadiego Mesa and Lastra Bello, 2015; Abbotts et al., 2022). However, some studies have failed to find evidence of an association between overweight or obesity and changes in CBD pharmacokinetics (Williams et al., 2021; Abbotts et al., 2022).

Metabolism: CBD undergoes extensive first-pass metabolism, with 85% being eliminated and converted into the active metabolite 7-hydroxy-CBD (Fiani et al., 2020). This process is mediated by cytochrome P450 enzymes CYP2C19 and CYP3A4. Due to its low bioavailability when CBD is administered orally, sublingual administration is preferred. Given its hepatic metabolism, potential drug-drug interactions with enzyme-inducing or enzyme-inhibiting medications must be considered. CBD exhibits 94% protein binding and displays non-linear kinetics at doses exceeding 64 mg/kg/day. The steady state is reached within 2–4 days of treatment (Landmark and Brandl, 2020; Schaiquevich et al., 2020).

Elimination: The amount of CBD excreted in urine and feces is considered minimal (approximately 12%) and varies depending on the dosage and the specific compounded formulation ingested (Landmark and Brandl, 2020; Malaca et al., 2021).

## 4 Pharmacological interactions

### 4.1 Interaction with clobazam

The most clinically significant drug interaction is with clobazam, a benzodiazepine commonly used in the treatment of LGS and other refractory epilepsies. CBD is a potent inhibitor of CYP2C19, the primary enzyme involved in the metabolism of clobazam (Geffrey et al., 2015). Co-administration of CBD can lead to a significant increase in plasma concentrations of N-desmethylclobazam, sometimes by as much as three-to five-fold (Geffrey et al., 2015). This may potentiate clobazam’s sedative effects, resulting in symptoms such as somnolence, fatigue, and nausea (Table 1) (Gaston et al., 2017; Landmark and Brandl, 2020; Savage et al., 2020; Gilmartin et al., 2021). Therefore, when initiating CBD in patients on clobazam, it is crucial to monitor for symptoms and signs of clobazam toxicity. A gradual reduction in the clobazam dose may be warranted, and therapeutic drug monitoring of clobazam levels can be a useful tool to guide dose adjustments.

**TABLE 1:** Adverse-drug reactions to cannabidiol (adapted from (Lattanzi et al., 2018; McNamara et al., 2020; Zilmer and Olofsson, 2021; Navarro, 2024).

Adverse-drug reaction	Relative frequency	Severity
Anorexia	20.1%	Mild
Diarrhea	18.2%	Moderate
Xerostomia (dry mouth)	30.1%	Mild
Somnolence/Drowsiness	24.5%	Mild
Fatigue	10.3%	Mild
Headache	1.3%	Moderate
Nausea	10.8%	Moderate
Allergy	2.6%	Severe
Increase in transaminases and bilirubins^a^	16.1%	Moderate
Thrombocytopenia^a^	10.3%	Severe

^a^ with a concomitant use of valproic acid.

### 4.2 Interaction with valproate

A pharmacodynamic interaction has been observed between CBD and valproate. The concurrent use of these two medications is associated with a higher incidence of elevated liver transaminases compared to either drug used alone (Devinsky et al., 2018b). Although the concomitant use of these drugs is generally not recommended, it may be considered in select cases, such as refractory generalized epilepsy. In such instances, close monitoring of serum valproic acid levels and liver function tests is essential (Table 1) (Navarro, 2023).

### 4.3 Other anti-seizure medications

CBD may also affect the metabolism of other ASMs, although generally to a lesser extent than with clobazam. It has been shown to increase levels of topiramate, rufinamide, zonisamide, oxcarbazepine, eslicarbamazepine, perampanel, and lamotrigine (Gaston et al., 2017; Socała et al., 2019; Arzimanoglou et al., 2020; Gilmartin et al., 2021). Interactions have also been reported with stiripentol, felbamate, phenobarbital, carbamazepine, and phenytoin (Gaston et al., 2017; Socała et al., 2019; Arzimanoglou et al., 2020; Gilmartin et al., 2021). In general, the concomitant use of CYP2C19 and CYP3A4 inhibitors may increase CBD serum levels, while enzyme inductors may reduce them.

Information from case series and mouse models studies suggests that CBD may increase the levels of brivaracetam, gabapentin, pregabalin, tiagabine; decrease the levels of levetiracetam; and leave unchanged the levels of lacosamide (Socała et al., 2019; Gilmartin et al., 2021). Furthermore, interactions between CBD and ethosuximide, ezogabine, fenfluramine and vigabatrin have not yet been evaluated (Gilmartin et al., 2021).

While routine therapeutic drug monitoring of all concomitant ASMs may not be necessary, clinicians should remain vigilant for signs of toxicity or reduced efficacy when CBD is introduced into to a patient’s treatment regimen.

## 5 Dosing considerations

The effective dosing of CBD in the treatment of DRE is a critical determinant of its therapeutic success and is guided by a principle of “start low, go slow.” Dosing in children is typically weight-based, whereas in adults, it requires careful titration to achieve an effective and well-tolerated dose (Navarro, 2023). Given the superior bioavailability of the sublingual route compared to the oral route, administering CBD solutions sublingually is the recommended strategy.

### 5.1 Initial dosing and titration

For the approved CBD oral solution, the recommended starting dose in pediatric patients with LGS, DS, and TSC is 2.5 mg/kg twice daily, for a total daily dose of 5 mg/kg/day. In adult patients, treatment may be initiated with 5 mg–10 mg of CBD per dose, taken twice daily. This initial low dose allows for the assessment of initial tolerability.

The dose is typically titrated upward to reach a maintenance dose. A common titration schedule involves increasing the daily dose on a weekly basis. In pediatric patients, further increases can be made in increments of 2.5 mg/kg twice daily (5 mg/kg/day), as needed and as tolerated. In adults, the dose may be increased by 10 mg every 12 h, up-titrated weekly (Navarro, 2023; Navarro and Pérez, 2024).

### 5.2 Maintenance and maximum dosing

The recommended maintenance dosage for children generally ranges between 10 mg/kg/day and 20 mg/kg/day, divided into two doses. Clinical trials have demonstrated that both 10 mg/kg/day and 20 mg/kg/day of CBD are effective in reducing seizure frequency compared to placebo (Devinsky et al., 2017; Devinsky et al., 2018a; Devinsky et al., 2018b; Thiele et al., 2018; Thiele et al., 2021). The choice between these doses often depends on balancing efficacy and the emergence of ADRs. In adult patients, the median daily CBD dose has been described in approximately 200 mg (equivalent to 3.7 mg/kg/day considering an average weight of 67 kg) (Navarro, 2023).

The maximum reported dose of CBD was 50 mg/kg/day (Devinsky et al., 2016), which may be considered when the potential benefits outweigh the risks of increased ADRs. Doses exceeding this level are not well studied and may be associated with a higher incidence of ADRs, including significant elevations in liver enzymes.

The correlation between oral CBD dose, serum CBD levels, and the response rate has been evaluated. The findings showed inter-individual variability between the CBD dose and its serum levels, as well as variability in the metabolite serum levels across different brands of CBD oil and types of oral formulations (Malaca et al., 2021; Williams et al., 2021; Abbotts et al., 2022).

## 6 Gradual dose titration: a cornerstone of therapy

Slow and gradual dose titration is a fundamental principle for the safe and effective use of CBD in DRE. Rapid dose escalation may increase the likelihood and severity of ADRs, potentially resulting in the premature discontinuation of a treatment that could otherwise be beneficial at a well-tolerated dose (MacCallum and Russo, 2018).

Slow titration allows the patient’s body to gradually acclimate to the medication, thereby improving tolerability. It also facilitates the identification of the minimal effective dose for each individual, minimizing the risk of dose-related ADRs. Furthermore, it enables close monitoring of drug-drug interactions and allows for timely adjustments to the doses of concomitant ASMs (Schaiquevich et al., 2020).

ADRs can occur at any point following treatment initiation and during the dose-titration period. Therefore, dose adjustments should be made gradually. Patient response is idiosyncratic; thus, a low dose may elicit an ADRs in one individual, while the same dose might produce no ADRs in another. As the likelihood of serious ADRs increases with higher CBD doses, the clinical goal is to use the lowest effective dose for each patient.

If a patient experiences troublesome ADRs, the titration schedule can be slowed, or the dose temporarily reduced, with a gradual increase once the side effects have subsided. This patient-centered approach to dose titration is crucial for promoting long-term adherence and optimizing therapeutic outcomes.

## 7 Adverse-drug reactions monitoring

ADRs to CBD have been reported in 40%–50% of patients, with some studies noting rates as high as 85% (MacCallum and Russo, 2018; Arzimanoglou et al., 2020). The potential for hepatotoxicity is a key safety concern associated with CBD therapy. As noted, the risk of elevated liver transaminases increases with higher doses of CBD and with concomitant use of valproate (Devinsky et al., 2018b). If transaminase levels rise to more than three times the upper limit of normal accompanied by elevated bilirubin, discontinuation of CBD should be considered. CBD treatment is not recommended for patients with a pre-existing hepatic impairment.

Additionally, cases of thrombocytopenia have been reported in pediatric patients previously treated with valproate, occurring in 9 out of 87 children at the initiation of CBD therapy (McNamara et al., 2020).

It is recommended to obtain baseline liver function tests, including transaminases, bilirubins, and alkaline phosphatase levels, as well as a complete blood count prior to initiating CBD therapy in all patients. Follow-up testing should be performed at 1, 3, and 6 months after treatment initiation, with more frequent monitoring advised for patients receiving concurrent valproate (Navarro, 2023).

As CBD crosses the placenta and is excreted in breast milk, its use is not recommended in pregnant women with epilepsy. In animal studies, fetal plasma concentrations of CBD reached approximately 10% of maternal concentrations (Schaiquevich et al., 2020).

## 8 Conclusion

The introduction of medicinal CBD has provided a valuable new therapeutic option for patients with DRE. As advantages, CBD has been shown to be an effective alternative in reducing seizure frequency in DRE; it has a therapeutic benefit attributed to its novel mechanism of action, which differs from conventional ASMs, which allows it to be used in combination with these drugs. However, this therapy may have some disadvantages; it may have different drug-drug interactions with ASMs, so its use requires careful patient monitoring, including liver function tests and potential dose adjustments of CBD and concomitant ASMs, to optimize the balance between efficacy and safety.

A thorough understanding of CBD pharmacology, along with adherence to a “start low, go slow” and highly individualized approach, is essential for clinicians. By carefully addressing these factors, healthcare providers can safely and effectively harness the therapeutic potential of CBD to improve the lives of patients with DRE. Ongoing post-marketing surveillance and real-world evidence will continue to inform and refine the optimal use of this emerging treatment.
